# Short-term meditation training alters brain activity and sympathetic responses at rest, but not during meditation

**DOI:** 10.1038/s41598-024-60932-8

**Published:** 2024-05-15

**Authors:** Anna Rusinova, Maria Volodina, Alexei Ossadtchi

**Affiliations:** 1 Center for Bioelectric Interfaces, HSE University, Moscow, Russia 101000; 2 Laboratory of Medical Neurointerfaces and Artificial Intellect, Federal Center of Brain Research and Neurotechnologies of the Federal Medical Biological Agency, Moscow, Russia 117513; 3 Artificial Intelligence Research Institute, AIRI, Moscow, Russia; 4 LLC “Life Improvement by Future Technologies Center”, Moscow, Russia

**Keywords:** Peripheral nervous system, Neuroscience, Consciousness, Physiology, Neurophysiology

## Abstract

Although more people are engaging in meditation practices that require specialized training, few studies address the issues associated with nervous activity pattern changes brought about by such training. For beginners, it remains unclear how much practice is needed before objective physiological changes can be detected, whether or not they are similar across the novices and what are the optimal strategies to track these changes. To clarify these questions we recruited individuals with no prior meditation experience. The experimental group underwent an eight-week Taoist meditation course administered by a professional, while the control group listened to audiobooks. Both groups participated in audio-guided, 34-min long meditation sessions before and after the 8-week long intervention. Their EEG, photoplethysmogram, respiration, and skin conductance were recorded during the mediation and resting state periods. Compared to the control group, the experimental group exhibited band-specific topically organized changes of the resting state brain activity and heart rate variability associated with sympathetic system activation. Importantly, no significant changes were found during the meditation process prior and post the 8-week training in either of the groups. The absence of notable changes in CNS and ANS activity indicators during meditation sessions, for both the experimental and control groups, casts doubt on the effectiveness of wearable biofeedback devices in meditation practice. This finding redirects focus to the importance of monitoring resting state activity to evaluate progress in beginner meditators. Also, 16 h of training is not enough for forming individual objectively different strategies manifested during the meditation sessions. Our results contributed to the development of tools to objectively monitor the progress in novice meditators and the choice of the relevant monitoring strategies. According to our findings, in order to track early changes brought about by the meditation practice it is preferable to monitor brain activity outside the actual meditation sessions.

## Introduction

Meditation is known to have a number of beneficial effects. Multiple studies suggest that meditation can lead to improvements in attention, concentration, creativity, and problem-solving^1^, and enhances the ability to implement proactive and reactive cognitive control processes^2^. Various meditation techniques were also shown to induce positive effects on memory, fluency and cognitive flexibility^3^. Meditation programs can moderately reduce negative dimensions of psychological stress^4^. It is known that experienced meditators in a resting state, unlike novices, are calmer and have improved emotional stability^5^, which is supported by the objectively measured brain activity. Meditators show decreased activity in the regions related to discursive thoughts and elevated activity in the regions related to response inhibition and attention^6,7^. There is also evidence of positive effects of meditation on physical health, such as beneficial impact upon cardiovascular functioning^8–10^. Meditation was shown to modulate inflammatory gene expression, improving microvascular function^11^, cell-mediated immunity and reducing biological aging effects^12^. Thus, meditation based interventions are promising avenues towards preventing a range of pathological conditions, improving depression symptoms^13^, reducing the risk of age-related neurodegeneration^14^ and cerebrovascular diseases.

In order to maximize the benefits of meditation, it has to be performed properly, which requires meditators to go through the appropriate training. One of the associated challenges lies in the absence of a clearly formalized success criteria of mediation to guide a training process. Objective monitoring and qualitative assessment of meditation training success is hurdled by the contradictory findings, even within the same meditation type^15–17^.

An additional problem, associated with tracking meditation experience, is the absence of data on the expected trajectories of the CNS and ANS activity indicators during the actual meditation process. The majority of studies report average measures of CNS and ANS activity indicators during meditation and contain almost no data regarding the trajectory of the meditation induced changes that could potentially be employed to objectively gauge the meditation experience. The few exceptions are the articles^16,18,19^.

A number of studies have dealt with investigating changes in brain activity that occur during meditation sessions. The simultaneous increase in the power of theta and alpha rhythms in different brain regions is considered a typical change during the practice of meditation and may indicate a state of relaxed alertness^15^. According to Lomas' review, mindfulness meditation has been shown in 18 studies to enhance alpha activity, and in eight studies to enhance theta activity during mindfulness meditation, in comparison with an eyes-closed resting state in experienced meditators. Only two studies report a greater beta rhythm amplitude during mindfulness meditation.

In terms of autonomic nervous system activity, there is mixed evidence on how meditation affects physiological arousal, blood pressure, and heart rate. Some studies indicate a relaxed state with lower physiological arousal^20^ and increased heart rate variability in both low and high-frequency bands during meditation^21^. However, certain meditation techniques can promote an active state, resulting in an increased heart rate and altered heart rate-respiration coherence^22^.

Given the described discrepancy, there is an open question whether using identical success criteria would actually produce the same positive effect in all users. The importance of using an individualized approach to meditation based on brain activity has been emphasized previously^23^. One recent study^16^ demonstrated that a group of experienced meditators involved in Taoist meditation appeared to be split in two almost equal subgroups, each with its own trajectory of changes in physiological parameters when performing the same Taoist meditation in the same conditions. The fact that only the experienced participants segregated into two approximately equal groups, prompted the authors to suggest the existence of two different physiological meditation strategies.

What about the novices? This is the group of people who are particularly in need of feedback regarding the quality of their meditation experience. How much of the training is needed before the objectively measured changes start to occur? Whether these changes differ across novice participants, and what is the proper strategy to track them? Should we focus on tracking only the CNS, ANS activity, or both indicators during the actual meditation experience or should we resort to observing the changes during the resting state condition? The attempt to answer these questions is the main motivation of the current study.

As a model we used audio-guided staged Taoist meditation. This type of meditation can be considered a form of mindfulness practice because it emphasizes being fully present in the moment and consists of several consecutive stages including relaxation, body scan, stopping of internal dialogue, visualization, and instructed breathing. Also, such an explicit split into different stages enables additional reference points for tracking the CNS and ANS activity indicators during the actual meditation process.

## Results

### EEG

#### Resting state

We utilized a cluster-based permutation test to investigate variations in EEG power during the resting state, across various frequency bands, before and after the intervention. First, the post-intervention average resting EEG power across the frequency bands for each channel were normalized to the corresponding pre-intervention values to determine the relative change. Such an analysis was performed for the eyes-open and eyes-closed resting state separately.

**Figure 1:**
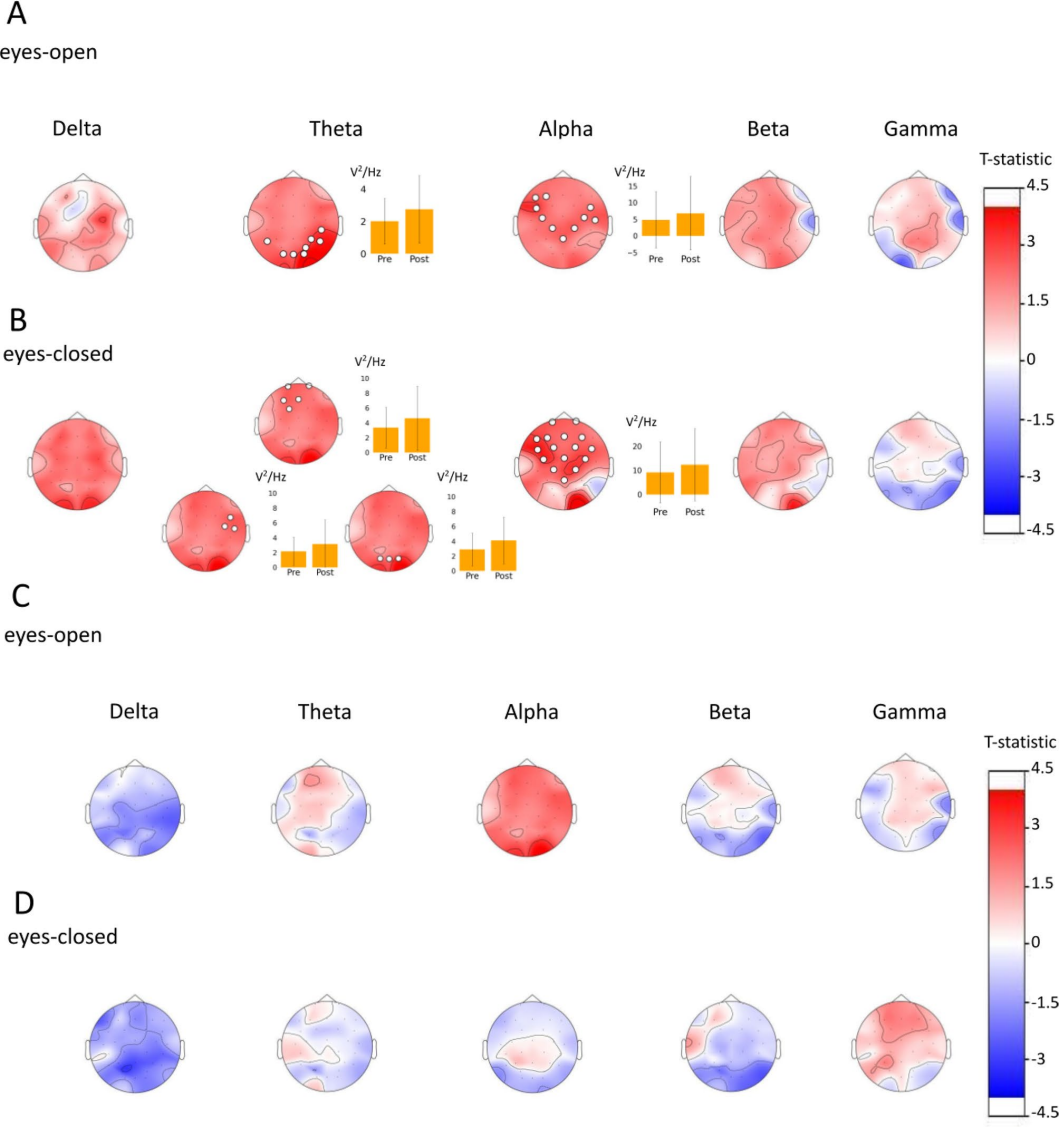
Analysis of variations in EEG power during the resting state across various frequency bands, before and after the intervention, using a cluster-based permutation test. The results are presented as topographic maps of the t-statistic. The upper panels (**A** and **B**) show the results in the meditator group. The lower panels (**C** and **D**) show the results in the control group. The channels comprising each statistically significant (*p* < 0.05) cluster are marked with white circles. For significant clusters, the barplots are presented showing the average spectral power density over that frequency band within the cluster $\pm{}$ the standard deviation before and after the intervention.

As shown in Fig. 1A,B our findings suggest that, primarily, the meditator group exhibited significant clusters, which may indicate changes in neural activity as a result of the meditation training.

Specifically, our findings demonstrate that meditators showed significant clusters in the theta band in the occipito-parietal area in the eyes-open condition (*p =* 0.007). Theta power averaged within this cluster exhibited an increase from 1.41 ± 0.678 in the pre-intervention resting state to 1.85 ± 1.172 after the training (z-statistic = 2.22, effect size = 0.758, *p =* 0.024, according to Wilcoxon test). In the eyes-closed condition we found three significant clusters. The first one was located in the frontal area (*p =* 0.019). The second cluster included three electrodes in the right parietal area (*p =* 0.026). The last one was located in the occipital area (*p =* 0.021). The Wilcoxon signed-rank test revealed increase of theta power after meditation training in all three of them (frontal cluster: increase from 3.36 ± 2.29 to 4.62 ± 3.77, z-statistic = 2.93, effect size = 1.0, p < 0.001; right parietal cluster: increase from 2.16 ± 1.44 to 3.15 ± 2.58, z-statistic = 2.67, effect size = 0.909, *p =* 0.005; occipital cluster: increase from 2.89 ± 2.29 to 4.11 ± 3.29, z-statistic = 2.76, effect size = 0.939, *p =* 0.003).

We also detected a big cluster comprising 10 electrodes located symmetrically in the central, left and right frontal areas (*p =* 0.002). This cluster exhibited an increase of alpha power in the post-intervention condition (4.8 ± 6.0 vs 6.8 ± 8.2, z-statistic = 2.93, effect size = 1.0, p < 0.001). In the eyes-closed state this cluster expanded and included the entire fronto-central area (*p =* 0.017; 9.16 ± 9.12 vs 12.35 ± 11.64, z-statistic = 2.31, effect size = 0.79, *p =* 0.019).

Post-intervention changes in EEG power in other frequency bands did not reach statistical significance based on the cluster-based permutation test.

Analysis didn’t find significant clusters in the control group.

#### Meditation

**Figure 2:**
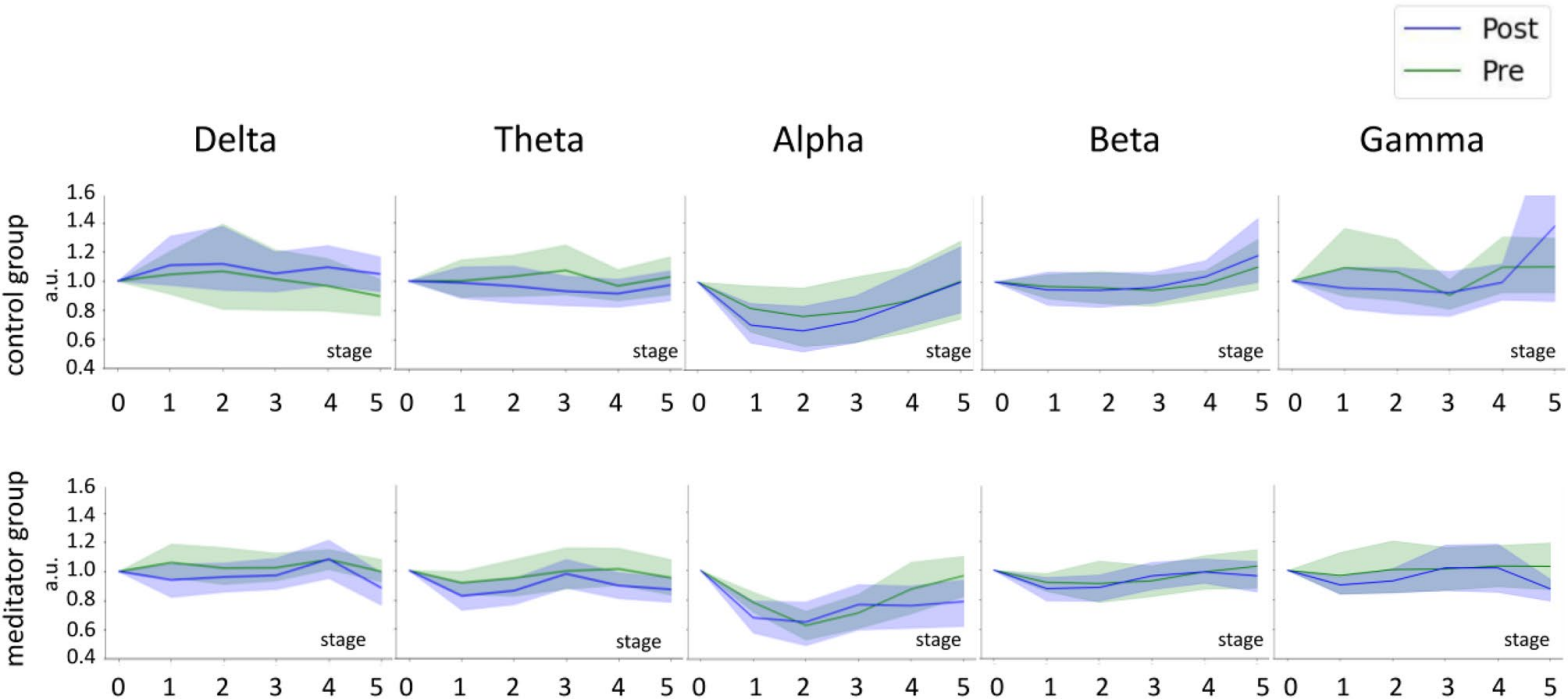
Changes in EEG power in the process of meditation compared to eyes-closed resting state averaged across all the channels. The green line marks the pre-intervention condition and the blue line marks the post-intervention condition. The data are presented as the mean ± 95% confidence interval. The x-axis corresponds to the meditation stages. Stage 0 corresponds to the eyes-closed resting state condition.

To investigate the effects of the intervention on the dynamics of power changes during the meditation process, a full-fledged space–time cluster-based permutation test was conducted. However, no significant clusters were observed. Although no difference between either two groups or pre- and post-intervention within a group was detected, we were still able to observe interesting, distinct and reproducible profiles of changes in EEG based band power indices for different indicators that we demonstrated in Fig. 2. In this figure we compared the average pre- and post-intervention values averaged across all electrodes in separate frequency bands. Before averaging, the values of each stage were normalized to the value at rest to show the changes of EEG power during meditation. For example, we could observe a clearly U-shaped profile in alpha rhythm dynamics, which coincides with results from Volodina et al., 2021^16^, observed in one of the meditator groups.

### Autonomous nervous system markers

#### Resting state. Eyes-open condition

**Figure 3:**
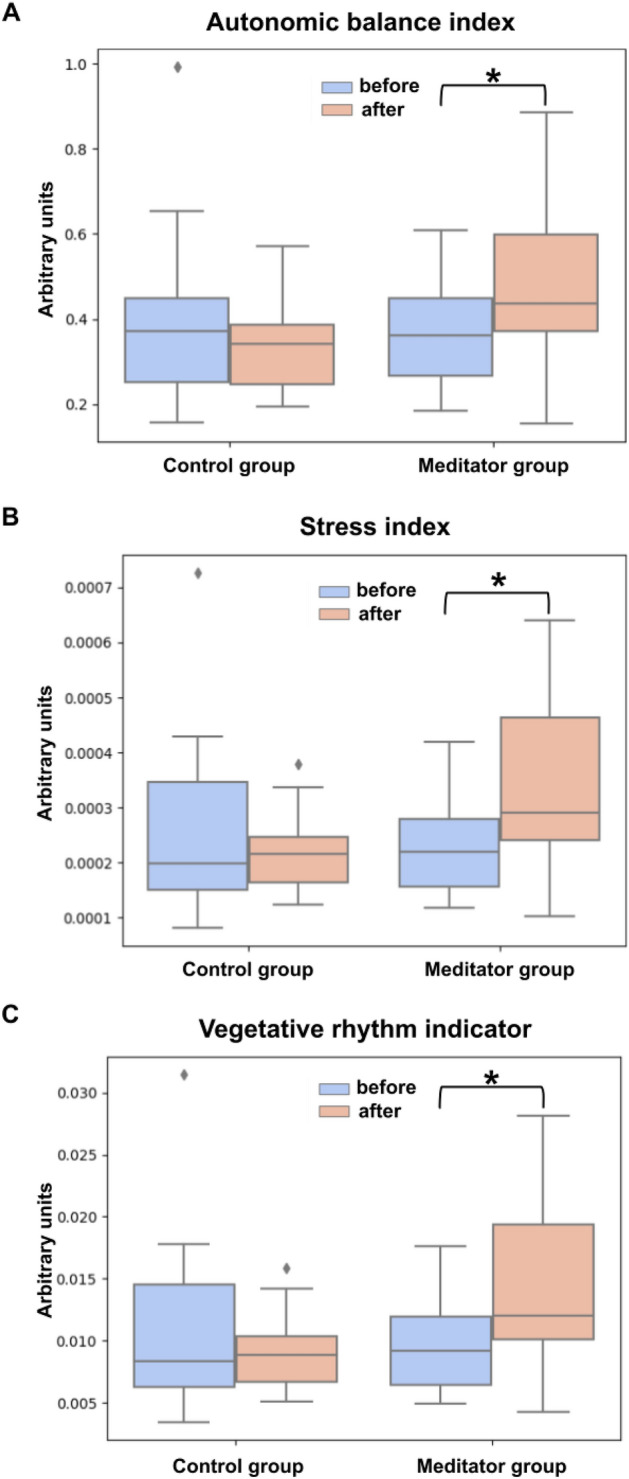
The differences between groups in PPG data during the eyes-open resting state (A is the Autonomic balance index, B is the Stress index, C is the Vegetative rhythm indicator). Data are presented as median ± interquartile range. * indicates significant (*p* < 0.01) differences between the meditator group and the control group according to the Wilcoxon signed-rank tests followed by FDR correction.

Using the Linear Mixed Effects model we found a significant interaction of the time point and group factors for the autonomic balance index (ABI), which indicates the ratio between the sympathetic and parasympathetic activity (estimate = 0.19, z = 2.90, *p =* 0.03), the stress index (SI), that reflects the stress of the cardiovascular system (estimate = 0.0002, z = 3.09, *p =* 0.028) and the vegetative rhythm indicator (VRI), which assesses the vegetative balance (estimate = 0.007, z = 3.08, *p =* 0.028). See “Materials and Methods” section for the mathematical expressions used to calculate these values and the appropriate references. All p-values were adjusted for multiple comparisons. The post-hoc Wilcoxon signed-rank tests revealed a significant increase in ABI (*p =* 0.003) SI (*p =* 0.003) and VRI (*p =* 0.004) in the meditator, but not in the control group. See the corresponding error-bars in Fig. 3.

#### Resting state.Eyes-closed condition

The Linear Mixed Effects model did not reveal significant interaction of time point and group factors for markers of the autonomic nervous system activity during the eyes-closed resting state, in either the meditator or control group. There was no interaction of group and time point factors for respiration rate and GSR during either the eyes-open or eyes-closed resting state as well.

#### Meditation

For PPG activity we did not observe any statistically significant interaction of “time point”, “meditation stage”, or “group”. However, we found a significant interaction of the time point and group factors for ABI (estimate = 0.14, z = 4.11, *p* < 0.001), Sl (estimate = 0.0001, z = 4.75, *p* < 0.001) and HR (estimate = 5.49, z = 2.77, *p =* 0.04). Analyzing “time point” effect separately in the groups, we found a significant effect of time point only in meditator on the HR (estimate = 5.82, z = 4.31, *p* < 0.001), ABI (estimate = 0.12, z = 5.22, *p* < 0.001), SI (estimate = 9.8*e−5, z = 5.93, *p* < 0.001).

We revealed an interaction between “group” and "time point” factors (estimate = 1.94, z = 2.68, *p =* 0.01), and “group”, “time point” and “meditation stage” for the number of spontaneous galvanic skin reactions (estimate = − 0.40, z = − 2,01, *p =* 0.09). However, a more detailed post-hoc analysis using the Wilcoxon test did not confirm a significant change in the GSR, in either the control or meditator group at any stage after the FDR correction. See table gsr_peaks_number_during_meditation.xlsx in OSF database (https://osf.io/zyt2u?view_only=e455f7e845f740d8b5d81b588f83c8aa).

There was also no effect of intervention on the respiration rate.

To summarize, we observed that after training, the meditator group showed a trend towards an increase in EEG power across a wide frequency range during meditation, but there were no significant changes in the dynamics of the EEG and PPG indices in the process of meditation after the course. We also did not find any division of the meditator group into subgroups with different changes in the EEG and PPG indices during meditation, either before or after the training.

## Discussion

In this study we focussed on the effects of meditation training in beginners, and thoroughly explored the changes in the ANS and CNS activity. The CNS activity was evaluated using electroencephalography (EEG), while a combination of respirometry (RESP), photoplethysmography (PPG), and electrodermal activity measurement was employed to assess the ANS activity. We aimed at simulating a real-life scenario in which contemporary urban inhabitants engage in meditation practices. The meditator group underwent a 16-session Taoist meditation training, each lasting an hour, over an 8-week period. The subjects were instructed not to practice meditation at home, to ensure the consistency of the intervention. It was important for the participants to receive offline training under the guidance of an experienced instructor, who had the ability to adjust the preparatory exercises and body position. Working with an experienced instructor minimizes the risk of undesirable consequences from meditation practice^24^. The control group spent an equivalent amount of time attending offline group sessions where the participants listened to audio books.

Overall, as revealed by our study,Taoist meditation training had a significant effect on the baseline physiological indicators during resting state intervals, but did not affect the meditation process itself. More specifically, the results of the study showed an increase in theta and alpha power during the eyes-open and eyes-closed resting state after the intervention in the meditator group but not in the control group. These could be interpreted as the signs of relaxed alertness state^15^ observed in the group of meditators. The results also showed changes in the heart rate variability indices associated with the increased sympathetic activity and alertness of the body (such as autonomic balance index, vegetative rhythm indicator, and stress index) during resting state with open eyes in the meditator group after the intervention.

The meditator group showed an increase in theta and alpha power during resting state. Such changes observed in the meditator group after the intervention are typical changes and have been previously reported in several studies of meditation^15,25^. One of the interpretations states that the increased alpha fluctuations reflect engagement in the internally directed tasks^26^.

In Takahashi's EEG based study, alpha power increased significantly during meditation, reflecting processes such as anticipation and attention^18^. More specifically, in our study, we observed increased alpha power in frontal, central, and parietal regions. It is known that frontal alpha activity can help to monitor and predict changes in some behavioral indices^27^ and the asymmetry of the alpha range of EEG in frontal areas may be related to emotional reactivity^28^. Based on the fact that high alpha activity in healthy individuals is negatively correlated with activation of the "negative emotion zone" in semantic emotional space^29^, we can then hypothesize that the increase of alpha band power in the resting state condition observed in meditators, may be indicative of an improved emotional state with longer meditation practice. Regarding cognitive processes, there are studies that confirm that it is the enhanced and synchronized alpha activity in the frontal area that is involved in higher brain processes during cognitive tasks^30^, higher alpha power in the right parietal cortex reflects focused internal attention during complex cognitive tasks, such as idea generation and mental imagery^31^. Also, alpha activity increases during working memory load^32^. Moreover, some studies have shown a correlation between the increased sensorimotor alpha rhythm power and bodily manifestations. For example, a study using biofeedback to reduce muscle tension found a link between increased alpha rhythm and muscle relaxation^33^. In other studies, increased cortical alpha rhythm accompanied more efficient sensorimotor interactions^34,35^. These findings, in combination with our observations, speak about the increased cognitive alertness and muscular relaxation in our meditators.

Theta power has been linked to attention and arousal^36,37^, memory^38^, affective cognitive processing mechanisms^39^, regulation of focused attention^40^, conscious awareness^41^, sustained attention, and mental effort^42^. In our study, we also observed increased theta power in the frontal, central, and occipital regions. Like increased frontal alpha, which may indicate increased cognitive load and improved psycho-emotional state, there are similar results related to increased theta power. Increased frontal theta activity is associated with increased effort to maintain high levels of performance^37^, mental tasks^43,44^, is related to attention concentration^45^, and may be a potential mechanism of cognitive control^46^. Frontal theta activity may be an EEG correlate of mood-related emotional processing^47^, associated with emotionally positive "blissful" experience and internalized attention^48^, and may be associated with reduction of anxiety symptoms in patients with generalized anxiety disorder^49^. As for other brain areas, it has been found that during cognitive tasks in the occipital region, the theta EEG frequency band exhibits increased power compared to baseline^50^. Theta band oscillations in the hippocampus were also shown to be crucial for temporal encoding/decoding of active neuronal ensembles and modification of synaptic weights^51^. The simultaneous increase of alpha and theta power has been associated with "relaxed vigilance"^52^. Studies have shown that the most reliable effects of a positive emotional state and internalized attention during meditation are reflected by increased local theta power and lower alpha power^48^. In another study, significant increases in theta and alpha power activity were found under meditation conditions when averaged across all brain regions, but in contrast to our results, alpha activity was found to be significantly higher in posterior compared to frontal regions^53^. Our results mostly corroborate findings reported by Nuhus et al.^54^, who found in the experimental mindfulness meditation group the meditation induced an increase of theta power in the right frontal and left parietal regions. This may be interpreted as the presence of a positive effect of meditation on the activation of memory related brain networks.

Our results also showed changes in heart rate variability indices known to be associated with increased sympathetic activity. The interplay of heightened alpha and theta power, along with increased sympathetic nervous system activity found in our study, may suggest a unique balance between alertness and relaxation in the participants. It is worth noting that there is inconclusive data from previous studies regarding sympathetic activation as a result of meditation training. There is a common belief that meditation leads to a decreased breathing frequency^55^ and the increased parasympathetic activity^18,56^, leading to relaxation. However, there is also evidence that meditation involves both sympathetic and parasympathetic systems, with a balanced coordination of both systems needed to maintain the meditative state. One of the studies revealed two different physiological strategies of meditation, characterized by distinct physiological changes^16^. The effects induced by meditation on the autonomic nervous system were found to depend on the meditation type^57–59^. Tang hypothesized that varying levels of meditative experience may result in different biological or physiological signatures, possibly involving the sympathetic system first and later the parasympathetic system^60^. Further research, with a longer meditation intervention, could cast some additional light onto this hypothesis.

Furthermore, we aimed to investigate the feasibility of identifying a personalized physiological strategy that could assist novice meditators in entering the meditative state. However, our analyses did not reveal significant differences in physiological measures changes during the meditation session. The trajectory of such changes was unaffected by the intervention. Thus, we could not make conclusions about the existence of a predisposition to certain physiological strategies of meditation, at least in the early stages of learning meditation. Based on our results, the 16 h of instructed meditation training over 8 weeks, appeared to be not enough for obtaining a stable physiological effect on the meditation process itself. This implies that longer intervention periods are needed to better understand the physiological effects of meditation and develop more effective meditation training methods. However, even such relatively short intervention, as used in our study, resulted in changes in the activity of the central and autonomic nervous system notable during the resting state. The elucidated, in this study, changes in the objective parameters of CNS and ANS functioning may indicate improved attention, particularly attention to internal cues, the rise of alertness and the increase of memory related brain networks activation.

In addition to the results presented in the manuscript, our statistical analysis revealed a trend towards an increase of delta range activity among meditators and a decreased delta band power in the control group. Such results are consistent with previous findings by Faber et al.^61^, who also reported an increase in post-meditation delta power. Many researchers, however, consider delta waves as a rhythm associated with cognitive processes^62^. Several studies have found links between delta activity and engagement in internal attention processes^63,64^ as well as with homeostasis and basal metabolic rate^65,66^. In our study, the trend of the increased delta rhythm power appears to coincide with sympathetic nervous system activation. We interpret this as signs of the activation of homeostatic and brain-body interaction processes in meditators. Tonic sympathetic activity has been shown to support resting metabolic rate in healthy adults in previous studies^67^, and increased brain-body interaction have been reported in the experienced meditators in several papers^68,69^.

Several limitations should be considered when interpreting the findings of this study.

Here, we have only investigated the effects of Taoist meditation training, and it is unclear whether these findings can be generalized to other types of meditation. Future research should explore the specificity of physiological changes in different types of meditation and investigate how Taoist meditation relates to other forms of this self-regulation practice. We have also limited our analysis to exploring the effects within each quantitative measure and did not examine the interplay between these measures, which constitutes an interesting and potentially fruitful future research direction. In this study, we did not use the specific questionnaires reflecting the success of mindfulness meditation such as MAAS^70,71^.

The additional important limitation of the present study is the modest sample size which may affect the validity of the conclusions, especially regarding negative findings, when no changes in regional EEG power were observed between the two groups of subjects. Where there were positive results, we have included effect sizes that appeared to be reasonably large and resulted in test power values on the order of 0.8, which we have also specified for each of the tests. In order to reassure our negative findings, additional experiments are required with a greater number of subjects. To conduct these and maintain the number of participants over a prolonged study duration, detailed questionnaires need to be completed, and significant monetary compensation has to be considered, to minimize the drop-out rate of subjects over the course of such a long experiment.

## Conclusion

The course of Taoist meditation, consisting of 16 h of training over 8 weeks, caused topically well organized changes in brain activity (increase in theta and alpha power), and markers of autonomic nervous system activity which indicate sympathetic activation. The effect was primarily observed in the resting state. There were no significant changes in the dynamics of indicators during the meditation session, which could be due to the insufficient duration of the training. Since we did not observe changes in the physiological indicators during the meditation process, we could not draw conclusions about the existence of a predisposition to one of the meditation strategies. Further, studies with increased duration of training are needed to answer this question.

As illustrated, during the meditation process we could observe clear stage-by-stage variations in the band power which forms frequency-specific oscillatory power profiles. However, we found no changes between these profiles for the experimental and control groups. This conceptually complicates the development of assistive devices aimed at “guiding” novice meditators during the meditation process. Accordingly, and based on our results, the focus in creating such digital assistants should be shifted towards monitoring neurophysiological activity during time intervals outside of the meditation session. As apparent from Fig. 3 these changes occur not only in the EEG derived parameters but are also detectable based on the markers of the ANS activity, which can be readily measured with a range of wearable devices, which renders hope for a rapid translation of our results into practical applications.

## Methods

### Subjects

The study included 28 participants who underwent pretesting, with 25 participants included in the final analysis. Three participants left the experiment due to the occurrence of undesirable side effects. The final sample included a 12-person group (ranging in age from 20 to 37 years, with three men and nine women and a mean age of 28.08 ± 5.45), and a 13-person control group (ranging in age from 21 to 38 years, with four men and nine women and a mean age of 27.69 ± 5.68). Inclusion Criteria: participants in both the experimental and control groups were selected from individuals aged 20 to 40 years. None of the participants in either group had any prior experience with meditation, and this criterion aimed to prevent any confounding effects from previous exposure. Also, participants should be without diagnosed mental illnesses or brain disorders and not taking drugs that affect the central nervous system, such as antidepressants or sedatives. Exclusion Criteria: inability to attend sessions and the occurrence of negative psychological effects (anxiety, problems, etc.) during meditation practice sessions. Before the experiment, we analyzed various physiological parameters, as well as gender and age to ensure the meditators and the control group were similar in all indicators. The experiment was conducted in accordance with the declaration of Helsinki. Participation in the study was voluntary. All participants provided written informed consent, approved by The HSE University Committee on Inter-University Surveys and Ethical Assessment of Empirical Research in accordance with the Declaration of Helsinki. All experimental protocols were approved by The HSE University Committee on Inter-University Surveys and Ethical Assessment of Empirical Research in accordance with the Declaration of Helsinki.

### Experimental protocol

**Figure 4:**
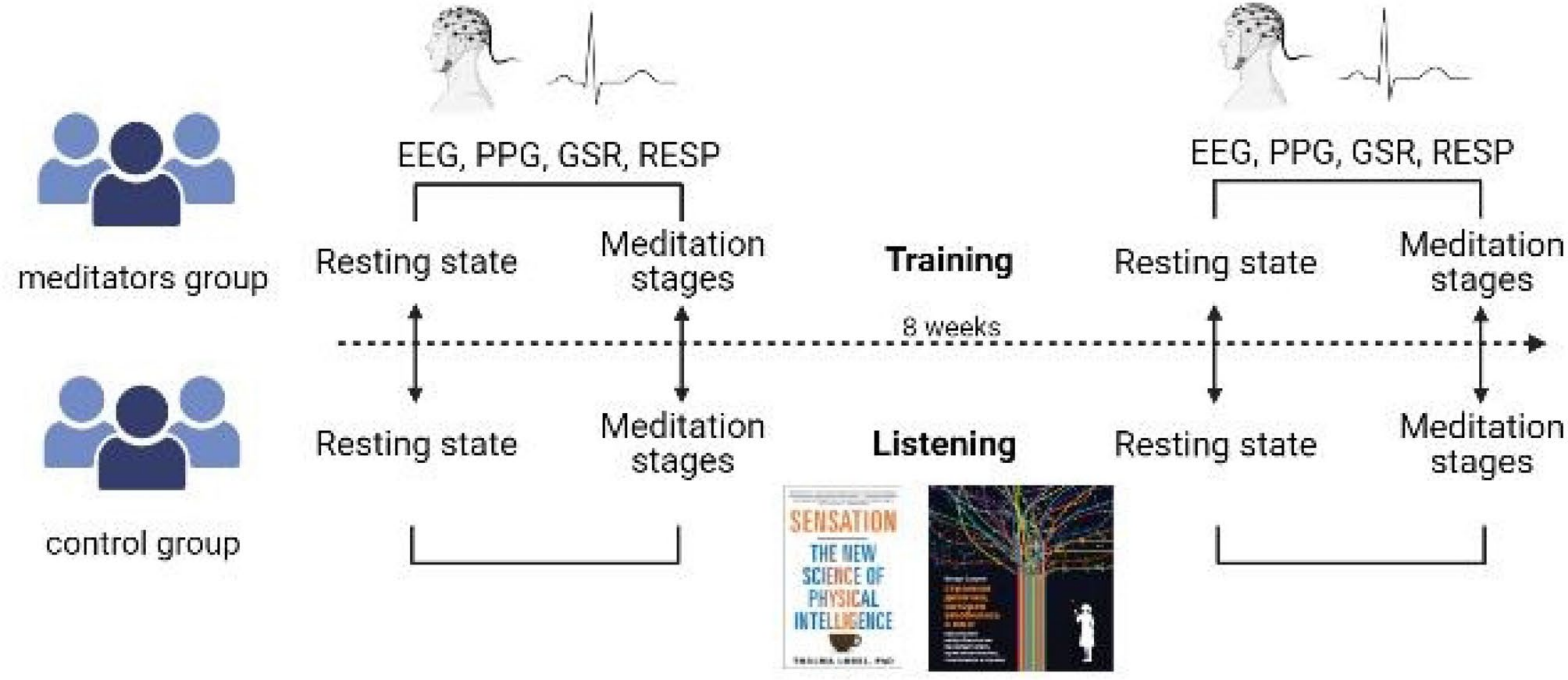
The design of the experiment with physiological testing, before and after the course/listening to audiobooks with an interval of eight weeks. The first instruction was used to collect baseline resting state data, immediately after was the full meditation session ( in ∼38 min). The testing included EEG, respiration (RESP), Photoplethysmography (PPG), Galvanic Skin Response (GSR).

The design of the experiment is shown in Fig. 4. For eight weeks, participants attended 1 h long group meetings twice a week, during which they engaged in a course of Taoist meditation with a qualified instructor (experimental group) or listened to audiobooks (control group, audiobooks: “A Warm Cup on a Cold Day—How Physical Sensations Affect Our Solutions”, Talma Lobel and “Healthy Brain, Happy Life: A Personal Program to Activate Your Brain and Do Everything Better”, Wendy Suzuki). Both groups took the course/lectures in the same auditorium, in the evening for 1 h, 2 days per a week. The meditation courses were on Tuesdays and Thursdays. The sessions with the control group were on Mondays and Wednesdays. The meditation lesson practically repeated the audio instruction used on the physiological testing (see Supplementary materials).

All participants were required to undergo physiological testing before and after the course. During such testing, the experimenter placed the EEG cap, PPG, GSR, and RESP sensors and measured physiological indicators during the resting state (4 min: 2 min eyes-open + 2 min eyes-closed) and during the Taoist meditation guided by audio instruction, delivered through the earphones (34 min).

Meditation protocol during testing. Prior to the meditation session, all the participants read the text of the meditation and could ask questions. The audio instruction was presented by an experienced meditation teacher and consisted of 16 stages. The end of each audio instruction marked the beginning of a new stage of meditation, and each stage lasted for about 2 min. For the subsequent statistical analysis we merged meditation intervals into the following 6 stages:

Resting state before meditation;

- Stage 1: Merged stages 1–4 (relaxation, body scan, taking position);
- Stage 2: Merged stages 5–7 (stopping internal dialogue);
- Stage 3: Merged stages 8–10 (visualization);
- Stage 4: Merged stages 11–14 (coming back, focus on breathing and body);
- Stage 5: Post Meditation resting stage.

The full text of meditation guidelines can be found in the Supplementary materials.

### Physiological measurement and equipment

#### EEG

The EEG data was recorded using a 30-channel wireless EEG system (SmartBCI, Mitsar, Russia) with a sampling rate of 250 Hz. The digital averaged ear signal served as a reference for all EEG data channels. The EEG data was precisely synchronized with the audio instruction. A Python script was used to simultaneously run the audio instruction and to collect EEG data.

### Photoplethysmography, Galvanic Skin Response, Respirogram

PPG, GSR, RESP signals were recorded using PolyRec (Medical computer system, Russia).

The PPG sensor was placed on the subject’s index finger of the right hand. The following filters were used: 4th order 0.5 Hz high-pass filter, 4th order 10 Hz low-pass Butterworth filter, notch filter at 50 Hz.

The GSR sensors were placed on the subject’s first phalanges of the ring and index fingers of the left hand. The following filters were applied to the GSR signals: 4th order 10 Hz low-pass Butterworth filter, notch filter 50 Hz.

The respirogram was measured with a thermometric sensor of nasal respiration placed under the nose, whose signal was filtered in the (0.05–10) Hz range using the 4th order Butterworth filter and a notch filter centered at 50 Hz.

### Data pre-processing

#### EEG

The EEG data underwent preprocessing, including bandpass filtering with a lower cut-off of 1 Hz and an upper cut-off at 40 Hz. A 50 Hz notch filter was also applied to suppress the powerline interference. Independent Component Analysis yielded 29 components, from which eye movement and muscular components were excluded. On average, five components were eliminated. Preprocessing was performed using NFBLab software^72^.

### Data processing

#### EEG

The EEG processing was performed the same way as in the previous Taoist meditation study^16^. The Welch method was applied to compute the EEG spectral power with 1 s Hanning window with 50% overlap. Scipy.signal.welch function was used. The power spectral density was then summarized by a simple averaging within the following frequency bands: Delta (1–4) Hz, Theta (4–8) Hz, Alpha (8–12) Hz, Beta (12–30) Hz, and Gamma (30–40) Hz.

MNE-python was used for all subsequent data processing.

#### PPG

Heart rate variability indices were analyzed in the same way as in the article^16^. Briefly, indices are defined as follows:

RR is the interval between successive PPG peaks, ME is the Median RR, AME is the amplitude of median, SD is the standard deviation.

1. The heart beats were detected with a commonly used peak detection algorithm implemented in routine^73^. The heart rate (HR) values were calculated using a 20-s long moving sliding window with a 5-s stride. Heart rate variability (HRV) analysis is one of the commonly used methods to appraise the activity of the sympathetic and parasympathetic nervous system.
2. HRV was calculated using pulse-to-pulse (RR) time series using a 60-s sliding window, with a stride of 30 s (30 s overlap). Then the obtained values within each meditation stage were averaged. The HRV-based metrics included the time domain and frequency domain indices.
3. RMSSD is the “sequential difference mean squared”, the square root of the mean of the squared successive differences between neighboring NNs (beat-to-beat intervals). High RMSSD values indicate high parasympathetic activation.
4. Autonomic balance index (ABI) indicates the ratio between the activity of the sympathetic and parasympathetic divisions of the autonomic nervous system^74^ (1).
  1 $$ABI=\frac{AME}{SD}$$
5. Vegetative rhythm indicator (VRI) assesses the vegetative balance (2). A lower VRI indicates greater activity in the parasympathetic nervous system, which suggests a shift towards a more balanced state of the autonomic nervous system^75^.
  2 $$VRI=\frac{1}{{\varvec{M}}{\varvec{E}}*{\varvec{S}}{\varvec{D}}}$$
6. Stress index (SI), which is a geometric measure of HRV that reflects the stress experienced by the cardiovascular system^76^ (3). High SI values indicate reduced variability and high sympathetic activation of the heart.
  3 $$SI=\frac{{\varvec{A}}{\varvec{M}}{\varvec{E}}\boldsymbol{*}100\%}{2\boldsymbol{*}{\varvec{M}}{\varvec{E}}\boldsymbol{*}{\varvec{d}}{\varvec{R}}{\varvec{R}}}$$

#### GSR

A zero-phase high-pass 4th order Butterworth filter with a cutoff frequency of 0.05 Hz was applied to the data to remove the slow trend and the number of spontaneous reactions was measured, defined as signal fluctuations with an amplitude greater than one standard deviation of the signal calculated for each individual subject during the entire recording. A sliding window with a length of 20 s with 5-s strides was used.

#### RESP

A peak detection algorithm was applied to the recorded breathing data. The respiratory rate was calculated using a moving window with a length of 20 s (strides of 5 s), all stages were then averaged. The respiration amplitude was calculated as the average difference between the upper and lower signal envelopes.

### Statistical data analysis

#### EEG

To determine the statistical significance of the changes in EEG power between the pre- and post-training conditions in the meditator and control group, we performed a cluster-level permutation paired t-test^77^.

The analysis was conducted using the mne.stats.permutation_cluster_1samp_test function with 1024 permutations. The average number of neighbors per electrode was 5.1, with a range from 3 to 8. We performed this test separately for the meditators and control groups. Average EEG power within the electrodes comprising significant clusters was calculated and the Wilcoxon signed-rank test was performed for obtained values to estimate the effect size.

During the analysis of EEG signal dynamics within the meditation process, all values were standardized to the baseline measurement obtained during eyes-closed conditions. We then examined changes in EEG power across various stages of meditation in comparison to the baseline. To determine whether the dynamics during meditation were influenced by the intervention, a cluster-based permutation test was employed.

#### PPG, GSR, and RESP data

Firstly, we analyzed the interaction of “group” (control/meditator) and “time point” (pre-/post-training) factors using the Linear Mixed Effects model^78^ for resting state data and interaction for “group”, “time point”, and “meditation stage” factors for meditation data. The analysis was performed utilizing the statsmodels.formula.api.mixedlm function. Fixed effects included group (meditator/control) and time point (pre-/post-intervention). Participant ID was included as a random effect to account for individual differences and minimize their impact on the results. For indicators with a significant interaction of factors after the FDR correction procedure^79^, the data before and after the intervention were compared using the Wilcoxon test. P-values obtained for the Wilcoxon test were also adjusted for multiple comparisons using the FDR correction procedure.

### Supplementary Information

Supplementary Information.

## Data Availability

Raw EEG, PPG, GSR, RESP data, supplementary tables and python code for this study can be found in the Open Science Framework webpage. See https://osf.io/3y4nj/?view_only=e455f7e845f740d8b5d81b588f83c8aa.
